# Molecular Dynamics and Nuclear Magnetic Resonance Studies of Supercritical CO_2_ Sorption in Poly(Methyl Methacrylate)

**DOI:** 10.3390/polym14235332

**Published:** 2022-12-06

**Authors:** Valentina V. Sobornova, Konstantin V. Belov, Alexey A. Dyshin, Darya L. Gurina, Ilya A. Khodov, Michael G. Kiselev

**Affiliations:** G.A. Krestov Institute of Solution Chemistry, Russian Academy of Sciences, 153045 Ivanovo, Russia

**Keywords:** supercritical carbon dioxide, poly(methyl methacrylate), nuclear magnetic resonance, molecular dynamics

## Abstract

The study of supercritical carbon dioxide sorption processes is an important and urgent task in the field of “green” chemistry and for the selection of conditions for new polymer material formation. However, at the moment, the research of these processes is very limited, and it is necessary to select the methodology for each polymer material separately. In this paper, the principal possibility to study the powder sorption processes using ^13^C nuclear magnetic resonance spectroscopy, relaxation-relaxation correlation spectroscopy and molecular dynamic modeling methods will be demonstrated based on the example of polymethylmethacrylate and supercritical carbon dioxide. It was found that in the first nanoseconds and seconds during the sorption process, most of the carbon dioxide, about 75%, is sorbed into polymethylmethacrylate, while on the clock scale the remaining 25% is sorbed. The methodology presented in this paper makes it possible to select optimal conditions for technological processes associated with the production of new polymer materials based on supercritical fluids.

## 1. Introduction

The study of intermolecular interaction structures in a medium with supercritical state parameters is a complex and multi-stage process where it is necessary to create new methods and improve known ones. The interest in this kind of research is due to both fundamental and practical components. Thus, the use of supercritical fluid technologies is one of the promising ways to create dosage forms with prolonged action as well as new modern medical materials with specified properties [1]. The use of supercritical fluid technology in the sorption processes by highly porous biodegradable polymer materials of medicinal compounds is one of the promising ways to create such medical materials. In such processes, one of the most common solvents is supercritical carbon dioxide (scCO_2_) due to the absence of surface tension and high penetrating power. However, the study of such processes is a difficult task since the sorption and swelling of the polymer are usually measured separately using different methods [2,3,4,5].

Currently, there are several approaches to the study of changes occurring with polymers during their interaction with supercritical fluids. These include optical reflectometry method [6], silica microbalance [7,8], vibrational spectroscopy [9,10,11,12,13,14,15] and some others [16,17,18,19,20]. However, most of these methods are adapted only for polymers in the form of films, and few studies are devoted to the study of powder in volume, which in most cases is close to the actual technological process of production. In this regard, the search for approaches to solving such problems is relevant. The research [21,22] demonstrates that nuclear magnetic resonance (NMR) spectroscopy on ^13^C nuclei is one of the most informative methods to study the kinetics of carbon dioxide (CO_2_) sorption into micropores of various aqueous calcium and sodium aluminosilicates of type X, Y, A. It is important to note that the results are presented in the bulk phase. It is logical to assume that this type of NMR experiment will be informative in the study of the kinetics of sorption of scCO_2_ into a polymer matrix. Pressure-controlled NMR spectroscopy is a promising method to study polymers in supercritical CO_2_ [23]. In addition, the chemical shift related to CO_2_ observed in the polymer-enriched phase can be used to estimate the amount of CO_2_ in this phase, which gives access to information about the behavior of polymers in CO_2_. In the case of block copolymers, NMR gives a unique idea of the supramolecular structure interfacial region of the core-shell type [24].

There are also many papers devoted to the study of various aspects of adsorption using NMR methods in organic solvents. NMR spectra can be recorded at precession frequencies of various nuclear, but ^13^C NMR spectroscopy is often preferable since it has a wide range of chemical shifts compared to ^1^H and, therefore, has a better resolution. Recently, nuclear magnetic resonance with pressure control has been used to study reactions and processes occurring in supercritical solvents [23,25,26]. However, there are no general methodological techniques described in the literature to study the processes of sorption and swelling of the polymer matrix in volume at supercritical solvent parameters at the moment.

We have developed and tested a method to obtain kinetic characteristics of polymer sorption and swelling in supercritical CO_2_ based on ^13^C NMR spectroscopy and molecular dynamic (MD) modeling. The approach presented in this paper is based on NMR spectroscopy methods applied to the assessment of changes in the characteristics of the polymer matrix in the scCO_2_ medium and provides a new way to measure CO_2_ sorption and swelling in the polymer volume simultaneously. Polymethylmethacrylate (PMMA) was chosen as a model compound for testing ^13^C NMR spectroscopy approaches. This choice is due to the large volume of experimental studies of the PMMA-CO_2_ system presented in the literature at various state parameters. In particular, in the papers [17,27] the authors studied the sorption of CO_2_ in PMMA at 32–65 °C and pressure up to 10 MPa. Kamiya investigated the sorption of CO_2_ in PMMA in the range of 35–200 °C and with pressure up to 6 MPa [28]. Rajendran and co-authors investigated the sorption behavior of CO_2_ in PMMA at 50, 65 and 80 °C and pressures up to 23.8 MPa [29]. Vogt et al. investigated the sorption of CO_2_ in PMMA 60 and 100°C and pressures up to 12 MPa [30]. Shie and Liu studied sorption and CO_2_/PMMA interactions at temperatures of 32, 42 and 52 °C and pressures up to 35 MPa [31]. It is worth noting that the NMR method is rarely used in practice due to the need to use isotope-labeled ^13^C samples. However, if a certain density of a supercritical fluid based on CO_2_ is achieved, the sensitivity of the ^13^C NMR experiment may be sufficient to register spectra with a good signal-to-noise ratio. This circumstance was the reason for the parameters of performing kinetic NMR experiments, namely *ρ*_CO2_ = 1.3*ρ_c_ (*ρ*_c_ = 467 kg/m^−3^) of critical density. On the other hand, the impregnation process should be sufficiently slow in the NMR time scale to be able to register the observed sorption and swelling effects based on ^13^C NMR spectroscopy. The feature of NMR approaches is that the time scales of the physicochemical processes studied are limited to minutes and hours. However, the initial stages of sorption processes occur at the peak and nanosecond scales. From this point of view, the NMR study will be well complemented by MD modeling.

It is good practice to use computer simulation approaches in addition to experimental techniques when investigating complex processes. In this work, the process of sorption of supercritical carbon dioxide into the matrix of poly (methyl methacrylate) is studied by the method of classical molecular dynamics. Although the time and length scales of MD simulations are limited to a few nanoseconds and nanometers and significantly differ from experimental scale, MD is a useful and powerful tool for explaining phenomena observed in the experiment and at the same time for investigating systems at the molecular level. Previously, systems containing PMMA and scCO_2_ were studied by MD [32,33,34,35], and it was shown that computer simulation allows obtaining molecular insight on swelling behavior of the polymer and obtaining tendencies sufficiently agreed with the ones observed in experiment.

## 2. Materials and Methods

### 2.1. NMR Experiments and Methodology

**Figure 1:**
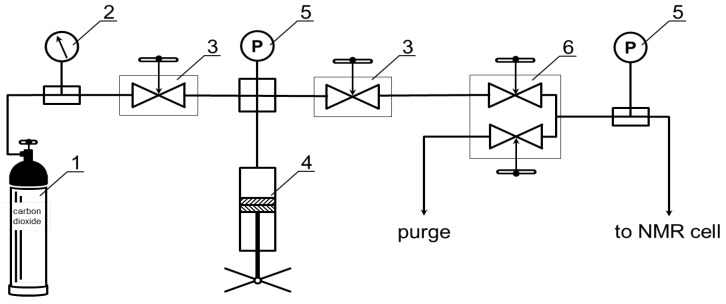
Scheme of the apparatus for producing and maintaining high-pressure for NMR measurements in carbon dioxide media, where 1 is a cylinder with carbon dioxide, 2 is a pressure gauge, 3, 6 are taper seal valves for filling (**upper**) and purging (**lower**), 4 is a manual press, 5 and 8 are electronic pressure transmitters, and 9 is a high-pressure NMR cell (see. Figure 2).

To measure the NMR spectra of ^13^C at supercritical parameters of the CO_2_ state, a system to create and maintain high pressure in real time was used (see Figure 1), based on a unique scientific installation “Fluid Spectrum” (https://ckp-rf.ru/usu/503933/ accessed on 6 December 2022) of G.A. Krestov Institute of Solution Chemistry of the Russian Academy of Sciences. The installation connects a high-pressure cylinder (Figure 1 pos. 1) containing carbon dioxide gas, with high-pressure NMR cell due to a high-pressure capillary. Filling and emptying high-pressure NMR cell is possible because of the system of taper seal valves (Figure 1 pos. 3, 6). Pressure monitoring is implemented using a pressure gauge (Figure 1 pos. 2) and electronic pressure transmitters (Gems™ Sensors&Controls, Basingstoke, UK) (Figure 1 pos. 5), a pressure adjustment in high-pressure NMR cell is carried out by means of a manual press (Figure 1 pos. 4). The accuracy of maintaining the pressure was ± 0.05 MPa.

**Figure 2:**
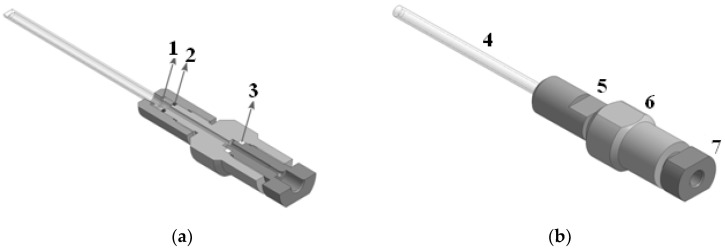
(**a**) High-pressure NMR cell (cross section): 1 is a capillary inlet seal (Teflon, Chemours, Wilmington, DE, USA), 2 is an ampoule seal (MVQ Silicones GmbH, Weinheim, Germany), 3 is a compensating gasket (Caprolon—polyamide, Shik Polymers, Moscow, Russia) (**b**) High-pressure NMR cell (full view): 4 is a high-pressure tube (synthetic single crystal sapphire Al_2_O_3_), 5 is a tube holder (D16T, Metatorg, Ivanovo, Russia), 6 is a input port (D16T, Metatorg, Ivanovo, Russia), 7 is a capillary inlet high-pressure (D16T, Metatorg, Ivanovo, Russia).

High-pressure NMR cell (Figure 2b pos. 4) [36,37,38] is a single crystal sapphire tube of high-pressure production (Daedalus Innovations, Aston, Pennsylvania, USA). The small size of the ampoule, which has an inner diameter of 3 mm, an outer diameter of 5 mm and a total length of 87 mm, allows it to work at pressures up to 30 MPa. The sapphire ampoule is equipped with an upgraded [39] cell fixation system (Figure 2b pos. 5), capillary mounts (Figure 2b pos. 6, 7) and sealing elements (Figure 2a pos. 1, 2, 3) to supply CO_2_ continuously. The temperature range of the cell is limited by the temperature range of the NMR spectrometer sensor.

NMR spectra were obtained using a Bruker Avance III 500 (Bruker Co., Karlsruhe, Germany) spectrometer equipped with a 5 mm Bruker TBI probe. The temperature was controlled by a VT-2000 prefix (Bruker Co., Karlsruhe, Baden-Württemberg, DE) with a Bruker BCU cooling unit (Bruker Co., Karlsruhe, Germany), the air consumption was 535 L/hour. Temperature calibration was carried out using a standard K-type thermocouple (Bruker Co., Karlsruhe, Germany). ^1^H NMR spectra of methanol were used as a method of temperature verification and calibration [40].

Polymethylmethacrylate (PMMA) powder was used as a sorbent (CAS No: 9011-14-7) of the Aldrich^TM^ (Sigma–Aldrich, Moscow, Russia). The impregnation was carried out in a carbon dioxide environment (CAS No: 124-38-9) of the primary standard, GOST 8050-85 (CO_2_—99.995%, H_2_O— < 0.001%) produced by Linde Group Russia (Balashikha, the Linde Group, RF). All substances were used without additional purification.

^13^C NMR spectra of scCO_2_ were obtained using a pulse program included in the software package of the TopSpin 3.6.1 for NMR spectrometer. The spectra were measured at a temperature of 50 °C and a pressure of 250 bar in the spectral range of 284 ppm to reduce the statistical error, 1024 scans were performed to obtain each spectrum, followed by averaging. A total number of 93 NMR ^13^C spectra were obtained with a fixed time interval between them equal to 90 min.

### 2.2. NMR Experiment Parameters Selection

To select the parameters of the experiment, we turned to the experimental data on the kinetics and sorption of PMMA in CO_2_, given in the literature [41]. As shown in [41] CO_2_ is better sorbed by the polymer matrix with increasing pressure. Thus, to increase the sensitivity of the ^13^C NMR experiment, a pressure over 20 MPa should be chosen. At the same time, the dependence of the sorption logarithms on pressure is demonstrated by the presence of two sorption modes [41].

The first sorption mode is observed at relatively low pressure up to 10 MPa and is characterized by low values of sorption intensity and capacity. The opposite mode is observed for pressure above 10 MPa and is characterized by relatively high values of capacitance and intensity, which makes it preferable for ^13^C NMR studies. However, when selecting the temperature values, some ambiguities arose. On the one hand, the sorption intensity in the high-temperature regime increases with increasing temperature, on the other hand, the sorption capacity decreases by an order of magnitude, which, of course, can significantly reduce the sensitivity in the ^13^C NMR experiment. With this in mind, when setting up the ^13^C NMR experiment, a temperature of 50 °C was used. It is due to a reduction in the duration of the NMR kinetics experiment from two weeks to one and the possibility of observing the process in a reasonable time. It is worth noting that the obtained parameters of in situ measurement of sorption and swelling in the volume of polymer powder can be used to optimize the production processes of polymer impregnation in supercritical liquids.

### 2.3. Computational Details/MD Method

**Figure 3:**
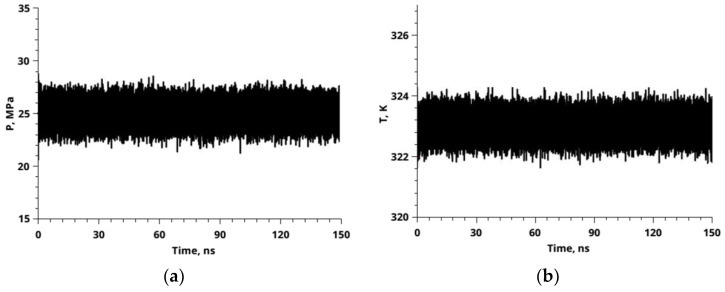
Time dependence of pressure (**a**) and temperature (**b**) during simulation process for 150 ns with a time step of 2 fs. These graphs confirm the reliability of the use of the barostat and thermostat and the absence of parameters of state (temperature in K and pressure MPa) changes throughout the duration of the simulation.

Each polymer chain first contained 100 monomers; then 108 chains were used to construct a three-dimensional structure with an initial density of 1000.0 kg/m^3^. The MD simulations were conducted in graphics processing unit-accelerated GROMACS v5.0.7 [42]. In our previous work [33], we described the methodology of bulk PMMA simulation with the Optimized Potentials for Liquid Simulations-All Atom (OPLS-AA) force field in detail [43]. In the current study, we used the same Lennard-Jones (LJ) and partial atomic charge parameters as in [33]. For carbon dioxide, we used the model developed by Z. Zhang and Z. Duan [44]. To calculate the cross site-site interactions, we used the geometric mean mixing rule for both LJ parameters. The temperature (323 K) and pressure (25 MPa) were controlled by a Nose’-Hoover thermostat [45,46] and a Parrinello-Rahman barostat [47], respectively. In Figure 3, time dependence of pressure and temperature during the simulation process is shown. The leap-frog integrator has been used for solving the equations of motion [48]. The cutoff radius was set to 1.5 nm for all the interactions. In the case of long-range electrostatic interactions, we used a particle mesh Ewald [49,50] with a grid spacing of 0.25 nm and an interpolation order of four. The constraints were implemented using the LINear Constraint Solver (LINCS) algorithm, to preserve the correct bond lengths [51].

A sample of PMMA (with linear dimension of about 16 nm) consisting of 108 chains equilibrated at 323 K and 0.1 MPa was placed in the center of a cubic cell with periodic boundary conditions and immersed in 242,522 CO_2_ molecules equilibrated in advance at 323 K and 25 MPa. After the energy minimization, we performed production run simulations in the *NpT* ensemble for 150 ns with a time step of 2 fs. Data for analysis were collected every 0.5 ps. The final length on the cubic simulation cell was 30.36 nm.

### 2.4. RRCOSY Analysis

The basic principle of using the ARCOS method is based on inverting relaxation curves with one or more relaxation distributions [52,53], according to Equation (1):

(1) $$L\left(g\left(-R_{n}\right)\right)=\int g\left(R_{n}\right)\exp\left(R_{n}t\right)dR_{n}$$

where *g(R)* is the relaxation velocity distribution function (*R_n_* = 1/*T_n_*) which is defined only for *R* > 0. In practice, when conducting NMR experiments, the inverse Laplace transform is used. In particular, the distributions of the inverse transformation *L*(*T*_1_) for the spin–lattice relaxation time *T*_1_ and *L*(*T*_2_) for the spin–spin relaxation T_2_, with experimental noise *E*(*t*), are determined by Equations (2) and (3):

(2) $$M_{SR}(t)=l(T_{1})\left[1-\exp\left(\frac{-t}{T_{1}}\right)\right]dT_{1}+E(t)$$

(3) $$M_{CPMG}(t)=l(T_{2})\exp\left(\frac{-t}{T_{2}}\right)dT_{2}+E(t{)}_{\;}$$

These equations are used to obtain one-dimensional distributions of relaxation times. In papers [54] two important features of conducting experiments with the Laplace transform were identified. The first feature is that the Laplace transform is noise-resistant; in other words, a small random error has little effect on the results after the transformation. The second feature is that the inverse Laplace transform is very unstable and needs additional regularization mechanisms. The description of algorithms for applying the Laplace transform to discrete data obtained during measurements is given in the papers [52,55].

It is worth mentioning the main feature to be characteristic of the inverse Laplace transform when constructing a two-dimensional correlation map of relaxation times. Distributions $1-\exp^{-t/T_{1}}$ and $\exp^{-t/T_{2}}$ may have several dependencies on the relaxation time in 2D NMR spectroscopy of the inverse Laplace transform which leads to a multidimensional spectroscopic image. In particular, the reverse Laplace transform spectroscopy used in this work *T*_1_–*T*_2_ Relaxation-Relaxation Correlation Spectroscopy (RRCOSY) [53,56] combines two pulse sequences. One of them is used by restoring the longitudinal magnetization after saturation of the NMR signal encoded in *τ*_1_, and the CPMG sequence (Carr–Purcell–Meiboom–Gill) encoded in *τ*_2_. Given the notation, the inverse Laplace transform in this case will take the form:

(4) $$M_{RRCOSY}(\tau_{1},\tau_{2})=\iint (1-\exp\left(\frac{-\tau_{1}}{T_{1}}\right)\exp\left(\frac{-\tau_{2}}{T_{2}}\right)L(T_{2},T_{1})dT_{1}dT_{2}+E(t)$$

where *E*(*t*) represents Gaussian additive noise.

Numerical integration according to Equation 4 is a certain transformation algorithm that requires constant adjustment of the selection of parameters, which in itself is a complex and time-consuming process. However, with the advent of the fast Laplace transform created by Venkataraman and co-authors [57], the condition has changed. Venkataraman’s algorithm significantly simplifies spectrum analysis and gives the opportunity to identify narrower areas of correlation. Since NMR relaxation processes are often first-order processes, it is the inverse Laplace transform (2D ILT) that translates data from the time domain of the experiment into the corresponding correlation spectra of relaxation time values. Information about whether there is more than one distinct mode or peak in two-dimensional relaxation time spectra can provide direct data on sorption sites and their number. In the literature, it has been shown [58,59,60,61] that time domain analysis of multidimensional 2D ILT relaxation experiments, such as RRCOSY, can quantify exchange parameters quite efficiently.

However, obtaining information about exchange processes by directly applying the inverse Laplace transform equation is often very difficult, since it is extremely sensitive to the initial experimental data in the case of two peaks in the 2D ILT spectra. In practice, there are two approaches to obtaining qualitative data for the selection of processing parameters based on the magnitude and shape of the peaks [62,63], one with additional modeling, and the other without it. Thus, in the approach adapted to describe the processes of polymer matrix sorption, a model of relaxation exchange main processes in the sample is proposed with subsequent adjustment of the parameters of this model to achieve the best match with 2D ILT data [60]. The minimum R^2^ factor of the least squares method is chosen as the criterion of compliance. Such a model is a special case of multi-node relaxation where only two nodes are considered. This model was first proposed and described in detail by McConnell in 1958 [64].

A two-node model can be obtained from a kinetic description of the change in the magnetization balance with time for a two-position exchange:

(5) $$d/dt\left[\begin{array}{c} M_{a}-M_{a}^{eq}\\ M_{b}-M_{b}^{eq} \end{array}\right]=\left[\begin{array}{cc} -R_{a}-k_{a} & k_{b}\\ k_{a} & -R_{b}-k_{b} \end{array}\right]\left[\begin{array}{c} M_{a}-M_{a}^{eq}\\ M_{b}-M_{b}^{eq} \end{array}\right]$$

where *R*_a_ and *R*_b_ are the proper relaxation rates, longitudinal or transverse for two sites (*a* and *b*), and *k*_a_ and *k*_b_ are the exchange rate coefficients that characterize the transfer from site *a* to *b* and from site *b* to *a*, respectively. *M_a_* and *M_b_* represent the magnetization of specific exchange nodes, and *M_a_^eq^* and *M_b_^eq^* provide the magnetization values of nodes that are in thermal equilibrium with the applied field.

The magnetization balance equation in formula (5) can be written in a more compact form:

(6) dM/dt=AM

where *M* is a two–element magnetization vector of nodes and the matrix *A* contains a combination of the corresponding relaxation and exchange coefficients:

(7) $$A=\left[R+K\right]=\left[\begin{array}{cc} -R_{a}-k_{a} & k_{b}\\ k_{a} & -R_{b}-k_{b} \end{array}\right]$$

The presented model describes the evolution of magnetization in the delay regions of the pulse sequence, taking into account the relaxation conditions for transverse or longitudinal processes, depending on the interval. The initial conditions for each interval in the pulse sequence are provided by radio frequency pulses preceding the evolution of magnetization. The fitting is performed by adjusting the parameters in the matrix A (Equation (7)) using the least squares method for the pulse sequence so that the model predictions coincide with the experimental data in the best way. The analytical solution of Equation 6 was obtained and described in detail for several 2D ILT pulse sequences, including for RRCOSY [58,59].

A useful result that follows from the solution of the presented equations is expressed in the ability to predict peak amplitudes of 2D ILT spectra using the so-called peak matrix P [59].

**Figure 4:**
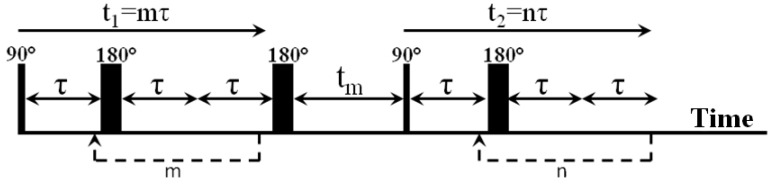
The RRCOSY pulse sequence used in the experiment consisting of two 90° and three 180° radio frequency pulses, where t_1_ is the time of the indirect encoding period, t_2_ is the time of the detection period, t_m_ is the mixing time, m and n are the numbers of echo times in the encoding periods.

The expression for matrices P for the RRCOSY pulse sequence (Figure 4) is written as:

(8) $$P_{T_{1}T_{2}}=2{\left[U_{2}^{-1}\exp(\left[R_{1}+K\right]t_{1})M(0)\right]}^{{}^{\circ}}{\left[1_{1\chi N}U_{2}\right]}^{T}$$

In expression 8, the eigenvectors U_1,2_ are a matrix of eigenvectors obtained from the matrix *R*_1,2_ + *K*, *M*(0) is the initial magnetization for the sequence, which in practice is usually equilibrium magnetization, t_1_ and t_m_ are given by the time parameters of the pulse delays, and 1χN is an N–element string vector. The symbol represents the element-wise multiplication (Hadamard multiplication) of the matrix between two multipliers. A detailed description and properties of this equation are presented in the paper [59].

Another method, the so-called data reconstruction method, aims to directly calculate the unknown function *L*(*t*_2_,*t*_1_) directly from the equation for *M_RRCOSY_*. At the first stage of *M*_1_ × *M*_2_ samples of *t*_1_ × *t*_2_ times and *N*_1_ × *N*_2_ samples of *T*_1_ × *T*_2_ relaxation times, problem (1) is discretized as:

(9) $$(K_{2}⊗K_{1})f+e=s$$

where $K_{1}\in R^{M_{1}\times N_{1}},K_{2}\in R^{M_{2}\times N_{2}}$ represent the discretized exponential kernels of the integral equation, and ⊗—binary product of matrices of arbitrary size (Kronecker product) [65,66], *s* ∈ *R^M^*^1·*M*2^—discrete vector of experimental noisy data, *f* ∈ *R ^N^*^1·*N*2^—distribution reordering vector, and *e* ∈ *R ^M^*^1·*M*2^—vector with sampled noise. Within this approach, the multiparametric Tikhonov regularization problem is numerically solved to minimize the functions of the type:

(10) $$\underset{f\geq l}{\min}\left\{\left.{\left\|(K_{2}⊗K_{1})f-s\right\|}^{2}+\sum_{i=1}^{N}\lambda_{i}{\left(Lf\right)}_{i}^{2}\right\}\right.$$

where ∥$(K_{2}⊗K_{1})f-s$∥—norm *L*_2_, *L* ∈ *R^N × N^*—discrete Laplace operator, *λ_i_*—regularization parameters, from *i* = 1, …, *Н*. This numerical solution is an iterative procedure where at each iteration step, suitable values of *λ_i_* are determined by imposing the condition that all nonzero products of *λ_i_* (*Lf*)^2^*_i_* have the same constant of values (Uniform PENalty principle) and an approximate distribution is obtained by solving an equation of form 10 using the Newton projection method. This approach was used in the work.

## 3. Results

### 3.1. Sorption of CO_2_ from NMR Experiment

Having determined the parameters of the ^13^C NMR experiment as 50 °C and 25 MPa, when developing the methodology for obtaining physico-chemical characteristics, it becomes necessary to find “markers” or “fingerprints” of the sorption and swelling processes of the PMMA system/CO_2_. It should be noted that ^13^C NMR signals of PMMA carbons are not observed in high-resolution NMR spectroscopy. This is due to the fact that without rotating the sample at a magic angle, the spectrum of a solid-state sample takes several hundred kilohertz and is leveled during mathematical processing of the spectrum. Thus, the value of the ^13^C chemical shift observed in the experiment for CO_2_ is an average sum of the adsorbed polymer matrix and free CO_2_. This picture is the opposite in comparison with the study of CO_2_ sorption by activated carbon fiber using ^13^C spectroscopy [67]. The overlap of the π-electrons of the carbon fiber leads to a sufficiently noticeable effect of screening the ^13^C nuclei for adsorbed CO_2_ molecules [68], which can be seen in the form of an additional wide signal with a shift to a strong field. It should be noted that such an effect is characteristic of activated carbon fiber, while for other systems, such as polymer matrices, in practice such changes in chemical shifts are not observed. Thus, the only possible way to obtain “markers” characterizing the processes of CO_2_ sorption into the polymer matrix of PMMA, as well as its swelling, is to change the spectral characteristics of one signal: resonance frequency, half-width at half-height and integral intensity.

To solve this problem, a series of ^13^C NMR experiments was carried out with subsequent processing of the spectral contour of the lines of resonant CO_2_ signals. A similar methodology is used for the analysis of vibration spectra [69]. In total number 93 ^13^C of the spectrum were obtained with a fixed time interval between them equal to 26 min. However, unlike vibration spectra, where the pseudo-Voigt function is used, in practice, the Lorentz function is used to process the spectral contour of the lines of resonant signals of ^13^C NMR spectra in liquids [70,71]. The main feature of the methodology used is that, unlike thin films, the work takes place directly with the powder, which is the best approximation to the conditions of polymer production.

**Figure 5:**
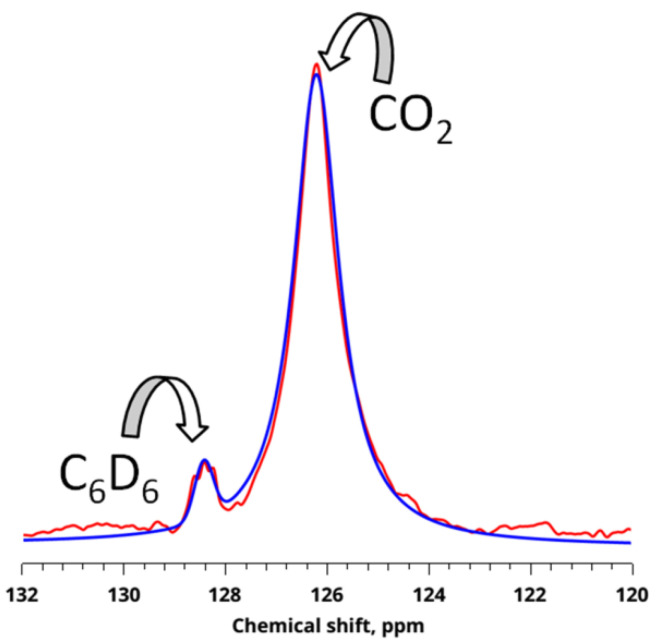
An example of quality of approximation of the spectral contour of the ^13^C NMR resonance signals of CO_2_ and standard С_6_D_6_ (red line) by Lorentz-like profiles (blue line) using pseudo-Voigt functions.

Figure 5 shows the ^13^C NMR spectra of free CO_2_ gas and CO_2_ adsorbed into the polymer matrix of PMMA at P = 25 MPa and T = 323.15 K. When analyzing the obtained ^13^C NMR spectra (Figure 5), only two signals can be seen. One of which is a triplet in a weak field and belongs to deuterated benzene sealed in an ampoule, used to calibrate the resonant frequency of 128.39 ppm, and the second located in a strong field is a singlet—CO_2_.

The magnitude of the chemical shift of the resonance peaks for CO_2_ in PMMA was the same as for the volume value (δ = 126 ppm) within the experimental error and the line full width at half maximum (FWHM) in these adsorbents (51 Hz ≤ FWHM ≤ 72 Hz) was slightly larger than for the volume (FWHM = 51 Hz in the volume of CO_2_). As discussed below, this effect is related to the peculiarities of sorption processes. Despite the fact that at the moment there are very few studies devoted to the sorption of CO_2_ into the polymer matrix by the ^13^C NMR method, there are several studies where the dynamics and local structure of several porous materials, such as zeolites, are analyzed [72] and activated carbon fiber [67]. As shown [22], for some zeolites, the difference in the magnitude of the chemical shift for the molecules of the bulk gas and adsorbed to the surface was 0.7 ppm in a higher field, which is also observed within the margin of error for activated carbon fiber. This suggests that the adsorbed CO_2_ molecule in the micropores of zeolites and fibers weakly interacts with the pore wall in these adsorbents. A similar situation was observed for our samples. The magnitude of the change in the chemical shift was only 0.2 ppm in the direction of a strong field, which indicates that PMMA is inferior in its sorption properties to zeolites or carbon fiber. However, the fact that changes in the chemical shift occur in the direction of a strong field also indicates that the change in the chemical shift of ^13^C CO_2_ signals in the gas phase is due to sorption processes into the polymer matrix. When studying sorption processes of CO_2_ into a polymer matrix, unlike carbon fiber, we do not observe broad signals in the 123 ppm region because the specific interaction of the surface of PMMA micropores and CO_2_ molecules with π electrons is much weaker compared to carbon fiber or absent altogether. For CO_2_ molecules enclosed in micropores, equilibrium has been achieved between adsorption and desorption of CO_2_ molecules. Therefore, by analogy with the results concerning the CO_2_ sorption of carbon fiber, it is logical to assume that the observed value of the chemical shift ^13^C for limited CO_2_ molecules is determined by the weighted average value of the chemical shift between CO_2_ adsorbed on the surface of the PMMA and CO_2_ co-existing in the micropore space and/or in the space near the entrance to the micropores.

**Figure 6:**
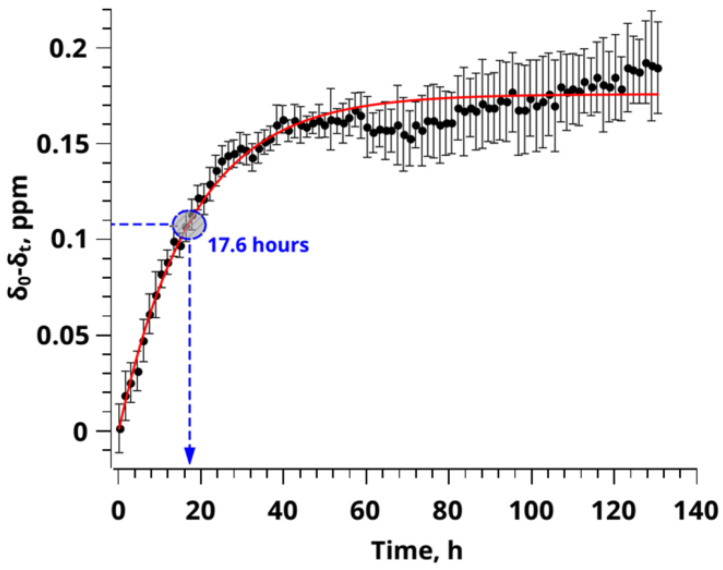
A graph of the change dependence in the parameter of the chemical shift of the ^13^C CO_2_ signal on time, approximated by a one-exponential model. Where δ_0_ is the value of the chemical shift of the ^13^C CO_2_ signal at the initial time, δ_t_ is the value of the chemical shifts of the ^13^C CO_2_ signal at observation time t. The blue circle on the graph indicates the correlation time of this sorption process, defined as the reciprocal of the rate constant of the sorption process (k).

Figure 6 shows a graph of the dependence of the chemical shift values of ^13^C NMR scCO_2_ on time. For clarity, dependence is presented in the hourly time scale. The chemical shift is responsible only for the sorption process, since during swelling the polymer does not change its magnetic environment and, accordingly, does not significantly contribute to the observed chemical shift of CO_2_. To describe the process of CO_2_ sorption in the pores of the PMMA polymer matrix, this dependence was approximated by the function that in practice proved to be the most accurate for describing the processes of CO_2_ sorption [73].

(11) $$\delta_{C}=\delta_{e}+\delta \exp(-kt)$$

where *δ_C_* is the value of the chemical shift of the resonant CO_2_ signal sorbed at time *t*, *δ_е_* is the value of the chemical shift of the resonant CO_2_ signal sorbed at saturation, *k* is the sorption constant, *δ* is the multiplier corresponding to the difference between the value of *δ_е_* and the initial value of *δ_C_* (at time *t* = 0).

As can be seen from Figure 6, the use of this mathematical model made it possible to approximate the experimental curve with high accuracy (R^2^ = 0.961). This confirms the predominance of the main process of CO_2_ sorption on the chemical shift value. The application of the proposed mathematical model made it possible to establish the rate constant of the sorption process (*k*), the value being 0.057 h^−1^, as well as the value of the equilibrium value of the chemical shift of 126.023 ppm (see Figure S1). The correlation time of the process was determined in accordance with the value of the rate constant *t_c_* = 1/*k* and was *t_c_* = 17.6 h.

**Figure 7:**
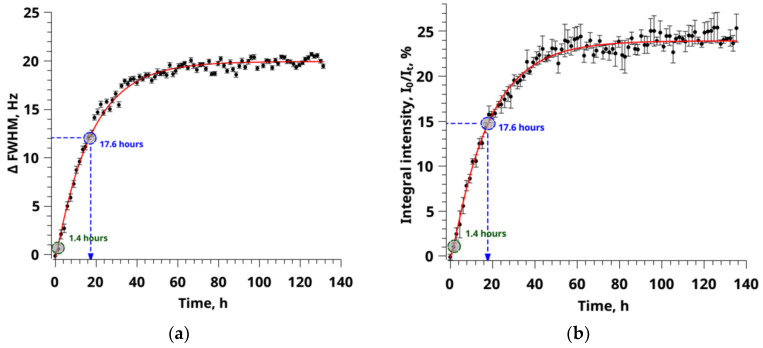
(**a**) A graph of the change dependence in the signal width parameter at the half-height of the ^13^C CO_2_ signal on time, approximated by a two-exponential model. Where ∆FWHM = FWHM_0_-FWHM_t_, where FWHM_0_ and FWHM_t_ are the half-widths of the signals at half-height at the initial time and observation time t, respectively. (**b**) A graph of the change dependence in the parameter of the integral intensity of the ^13^C CO_2_ signal on time, approximated by a two-exponential model. Where I_0_ and I_t_ are the values of the integral signal intensity at the initial moment of time and the observation time t. The blue and green circles on the graphs show the values of the correlation times of the sorption and swelling processes, respectively.

Using the values of the half-widths and integral intensities, it is possible to construct a kinetic dependence to determine the sorption parameters (Figure 7a,b). However, unlike the chemical shift, the integral intensity and half-width values can be influenced by other kinetic processes occurring with the polymer matrix during sorption. According to the literature data, such a process in the PMMA scCO_2_ system is swelling [20,74]. To test the hypothesis about the possibility of sorption processes in the analysis of kinetic dependences of half-widths, two mathematical models were used, by analogy with Equations (12)–(13).

(12) $$\Delta_{C}=\Delta_{e}+\Delta \exp(-k_{}t)$$

(13) $$\Delta_{C}=\Delta_{e}+\Delta_{1}\exp(-k_{1}t)+\Delta_{2}\exp(-k_{2}t)$$

Applying the equations, a fairly accurate approximation of the experimental data of the theoretical curve of values R^2^ = 0.985 was obtained. Moreover, these velocity constants and correlation times are somewhat overestimated compared with the values obtained from the analysis of chemical shifts. However, the use of a two-exponential model allowed not only to improve R^2^ = 0.986 but also to confirm the existence of the second faster process. Its speed is an order of magnitude higher than the sorption rate of 0.714 h^−1^ and the correlation time is an order of magnitude less than *t_c_*= 1.4 h, respectively. Moreover, the advantage of the proposed data analysis is the use of the values of constants obtained from the analysis of chemical shifts to approximate the data of integral intensity and half-widths in a two-exponential model. This method is possible due to the different nature of the parameters observed in the experiment.

Even though, at first glance, the approximation by a two-exponential model does not show significant improvements, this method allows us to capture such a slight effect of the polymer matrix swelling on the observed experimental parameters (Figure 7). The approach of analyzing experimental data by approximating mathematical models has a significant drawback, i.e., it is impossible to obtain unambiguous experimental evidence of the existence of more than one sorption process from a direct ^13^C NMR experiment.

### 3.2. Sorption of CO_2_ from NMR Experiment—2D RRCOSY

It is impossible to isolate the characteristic type of molecules by analyzing only the values of chemical shifts or integral intensities of ^13^C CO_2_. For further analysis of the results obtained, additional methods are required that can be used to justify the choice of a kinetic model to interpret experimental data. The analysis of the literature has shown that at the moment there is no reliable, universal technique to study the adsorption characteristics of a polymer matrix with a saturated fluid. In addition, sorption measurements based on chemical shift analysis will be very limited in terms of multicomponent systems inside polymer pores due to the wide overlapping lines associated with each component. The approach proposed in this paper is based on the analysis of the characteristic relaxation times of NMR. This approach is spared from these kinds of problems. Moreover, as shown in [75], the ratio of spin–lattice to spin–spin relaxation times (T_1_/T_2_) is directly related to the activation energy of desorption. Therefore, when developing this method, relaxation times were taken as the main parameters of the sorption characteristic.

One of the effective methods for distinguishing the types of molecular lability of CO_2_ in a material including in a polymer matrix is RRCOSY spectroscopy. The RRCOSY technique makes it possible to observe correlations of T_1_-T_2_ relaxation times by means of a two-dimensional inverse Laplace transform. The data obtained using RRCOSY spectroscopy represent populations of certain amplitudes which are generated due to various physical regions of mobility in the material and form regions (sites).

**Figure 8:**
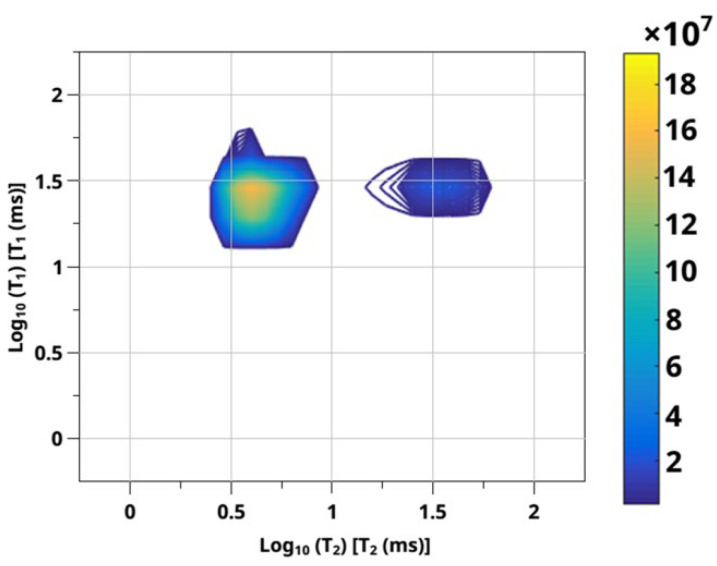
Two-dimensional correlation map obtained on the basis of the developed method. The ordinate and abscissa show the values of the logarithms of the relaxation times T_1_ and T_2_ spin–lattice to spin–spin relaxation times, respectively, while the sites in the spectroscopic image correspond to free solvent scCO_2_ (**left**) and impregnated into the polymer matrix (**right**).

Based on Venkataraman’s work on the direct model (in Section 2.4), proprietary algorithms were implemented that allow not only to build a spectrum but also to integrate the obtained correlation regions. The two-dimensional correlation map obtained based on the developed method is shown in Figure 8.

Figure 8 shows that two sites are present on the graph and although two T_2_ values have been identified, only one T_1_ value is observed. Two-dimensional ILT, predictable by the elements of the matrix of the analytical model [76], demonstrates four peaks of different amplitudes corresponding to the eigenvalues of the solution matrix. It corresponds to two peaks “diagonally” and two peaks of exchange. Diagonal peaks represent internal relaxation and internal relaxation weighted by exchange, which corresponds to the peaks with the shortest T_1_, T_2_ and the longest T_1_, T_2_ at the bottom left and top right, respectively. The other two peaks are exchange peaks: with a long relaxation time T_1_ which is associated with a short relaxation time T_2_, and a corresponding short time T_1_ associated with a long T_2_. The results of the 2D ILT experiment clearly show two main peaks: one is located at the longest values T_1_ and T_2_, and the second is the exchange peak located at the long values T_1_ and short T_2_. At the same time, the other two peaks are not visible. The presence of an exchange peak indicates that mixing between the populations of spin states occurs on the time scale of longitudinal relaxation in the system under study [77,78,79,80]. The exchange process averages T_1_ relaxation processes. Consequently, the internal values of T_1_ are difficult to isolate, and one T_1_ value is observed. However, it is still possible to provide internal T_2_ values using the direct data analysis method. Using a specially developed program that allows the construction and numerical integration of the obtained 2D ILT spectra, the values of integral intensities were obtained, and they amounted 0.15 to 14.72. Thus, the integral intensity of the non-diagonal peak is lower than the diagonal one. To interpret the results from a physicochemical point of view, it is necessary to understand that sites are physical areas of different mobility of scCO_2_ in the material to manifest themselves in a population of a certain amplitude and intensity. These two-dimensional experiments provide information about diffusion and trends in relaxation times which allow for a better understanding of the scCO_2_ state in the polymer matrix. It is worth noting that one of the populations, which manifests itself as a non-diagonal peak, is located within the parity line T_1_ = T_2_, where data representing sites of the free solvent scCO_2_ may appear. It is known from the literature that the farther the population is from the parity line, the more the rotational mobility of molecules is limited [56], and the closer the sites are to the parity line, the freer the rotational mobility. Thus, there are two pronounced sites in the polymer matrix that are physically responsible for free CO_2_ molecules and impregnated into the polymer matrix, numerical integration of these sites showing a significant excess of the concentration of CO_2_ in the polymer matrix over the free ones.

### 3.3. Sorption of CO_2_—MD Simulation Results

Different mobility of carbon dioxide molecules is also observed in the simulated system. Due to carbon dioxide actively interacting with the PMMA functional groups [33], the diffusion of the CO_2_ molecules inside the polymer matrix decreases compared to the pure fluid. Obtained data show that the diffusion coefficient of the CO_2_ molecules which are located inside the PMMA sample near the ester groups is more than 10 times lower ((2.74 ± 0.31) × 10^−5^ cm^2^/s than in bulk supercritical carbon dioxide (29.17 ± 0.47) × 10^−5^ cm^2^/s).

**Figure 9:**
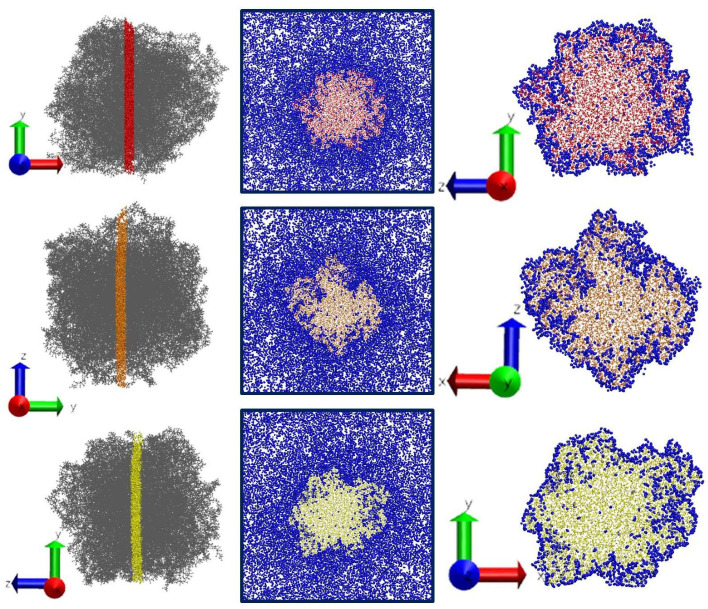
Instant snapshots of PMMA samples in three directions. Blue represents CO_2_ molecules. The red, orange, and yellow colors in the figure represent sections of the polymer from the center of the sample in three different planes. It can be seen from the figure that some of the CO_2_ molecules are not uniformly distributed in the polymer volume. The simulation results were used to further estimate the proportion of CO_2_ sorbed by the PMMA polymer matrix.

Figure 9 illustrates the instant snapshots of the simulated system final configuration. To better understand the penetration process of CO_2_ into the polymer we cut a 1 nm thick polymer layer from the center of the sample in three planes.

It can be seen from Figure 9 that by the end of simulation only a small part of CO_2_ molecules achieved the center of the PMMA sample and solvent distribution inside the polymer is uneven. Hence, we estimate the amount of solvent sorbed by the polymer per unit mass of the polymer in two ways. First, we calculated the amount of CO_2_ absorbed by PMMA taking into account not only molecules inside the polymer matrix but also molecules adsorbed on the polymer surface. The calculation was carried out as follows: the mass of carbon dioxide molecules, located at a distance of 0.35 nm from any PMMA atom was calculated for final system configuration, i.e., after 150 ns simulation. The criteria for such distance between solvent atoms and the polymer atoms was chosen based on the location of maxima on the atom-atom radial distribution functions CO_2_-PMMA and average interatomic distances obtained by classical and ab initio molecular dynamics in our previous work [33]. Then we used the equation:

(14) $$\alpha =\frac{m_{CO_{2}}}{m_{PMMA}}$$

where *m_PMMA_* is the mass of the polymer and *m_CO_*_2_ is the mass of sorbed solvent.

**Figure 10:**
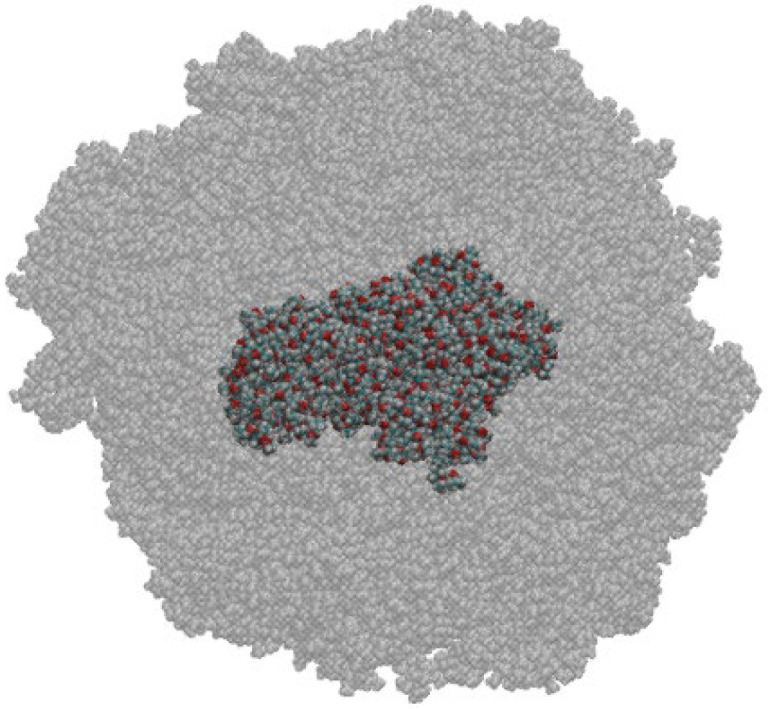
Snapshot of PMMA (gray) and the central part of the PMMA considered for calculation of absorbed CO_2._ This snapshot shows the volume that was used to calculate the parameters of sorption according to Equation 14 at various simulation times.

In the second case, we consider a few PMMA chains in the center of the polymer sample and in the same way estimate the amount of CO_2_ absorbed by the polymer per unit mass of the polymer. The part of the polymer considered for calculation *α* is presented in Figure 10.

The swelling degree calculated according to the first method, taking into account surface adsorption turned out to be 0.467 g/g. The amount of CO_2_ absorbed by the bulk phase of the polymer is 0.118 g/g.

## 4. Conclusions

The work is a comprehensive study of the sorption process of supercritical carbon dioxide PMMA at 50 °C and 25 MPa by NMR spectroscopy and classical molecular dynamics. For the first time, the method based on the joint use of two approaches (2D ILT and ^13^C NMR kinetic curve analysis) has been developed, which allows obtaining unique information about the state of CO_2_ in a polymer matrix, which is a useful scientific tool for the study of such systems. In the future, this approach can be developed to control the impregnation of medicinal compounds into a polymer matrix. According to the results of the ^13^C NMR experiment, it was found that the amount of sorbed scCO_2_ on an hourly scale was 24.9%. At the same time, the total amount of sorbed CO_2_ for all time according to RRCOSY is 98.1%. According to the molecular dynamics data, the amount of scCO_2_ sorbed by the polymer after 150 ns is 46.7%, taking into account surface adsorption. By the end of the simulation, the amount of carbon dioxide in the polymer volume was equal to 0.118 g/g. Thus, comparing the data obtained using the NMR experiment and computer modeling, we can conclude that on the second scale it is 26.5%. Much more important is that the combined use of NMR and MD modeling methods is a powerful tool for obtaining data on the sorption of CO_2_ into a polymer matrix.

## Data Availability

Not applicable.

## Supplementary Materials

The following supporting information can be downloaded at: https://www.mdpi.com/article/10.3390/polym14235332/s1, Figure S1: The ^13^C NMR spectra initial time (**a**) and after completed (**b**) sorption process of CO_2_ into the PMMA.
