# Supplementary material for: Molecular Dynamics and Nuclear Magnetic Resonance Studies of Supercritical CO2 Sorption in Poly(Methyl Methacrylate)
Source: Polymers (Basel). 2022 Dec 6;14(23):5332. doi: 10.3390/polym14235332 (PMC9737377; doi:10.3390/polym14235332)
Supplement: Supplementary file 1 [file polymers-14-05332-s001.zip › polymers-2056818-supplementary.pdf]

polymers

MDPI

Supplementary Materials

Molecular Dynamics and Nuclear Magnetic Resonance Studies of Supercritical CO_2 Sorption in Poly(Methyl Methacrylate)

Valentina V. Sobornova, Konstantin V. Belov, Alexey A. Dyshin, Darya L. Gurina, Ilya A. Khodov * and Michael G. Kiselev

G.A. Krestov Institute of Solution Chemistry, Russian Academy of Sciences, 153045 Ivanovo, Russia
* Correspondence: iakh@isc-ras.ru

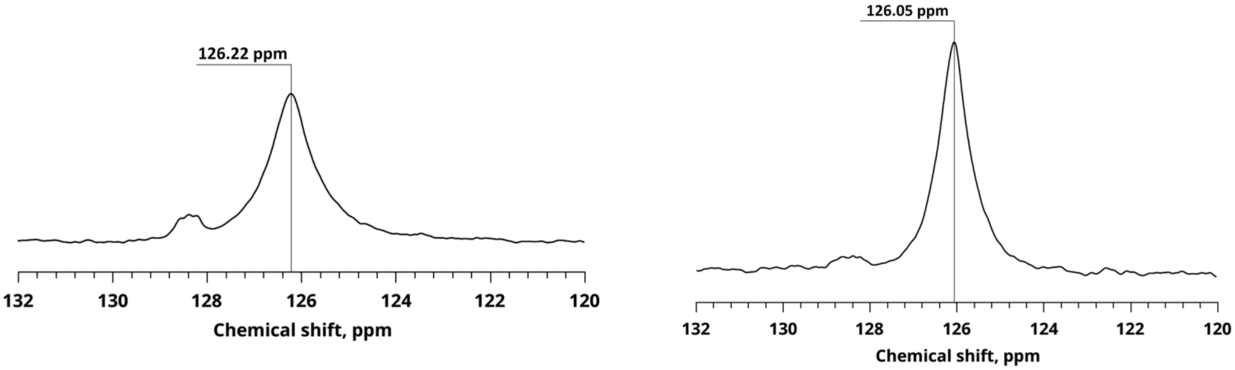

(**a**) (**b**)

Figure S1. The ^{13}C NMR spectra initial time (**a**) and after completed (**b**) sorption process of CO_2 into the PMMA.
